# Genetic analysis of tropical quality protein maize (*Zea mays* L.) germplasm

**DOI:** 10.1007/s10681-017-2048-4

**Published:** 2017-11-03

**Authors:** Susan G. Njeri, Dan Makumbi, Marilyn L. Warburton, Alpha Diallo, MacDonald B. Jumbo, George Chemining’wa

**Affiliations:** 1 International Maize and Wheat Improvement Center (CIMMYT), P.O Box 1041-00621, Nairobi, Kenya; 2 USDA ARS Corn Host Plant Resistance Research Unit, Box 9555, Mississippi State, MS 39762, USA; 3 Guinee-Semences, Immeuble Guinomar, Camayenne, Corniche Nord, BP 5603, Conakry, Guinea; 4 Department of Plant Science and Crop Protection, University of Nairobi, P.O. Box 29053-00625, Nairobi, Kenya

**Keywords:** Drought stress, Nitrogen stress, Genetic diversity, Predicted performance, Single nucleotide polymorphisms

## Abstract

Maize (*Zea mays* L.) is an important source of carbohydrates and protein in the diet in sub-Saharan Africa. The objectives of this study were to (i) estimate general (GCA) and specific combining abilities (SCA) of 13 new quality protein maize (QPM) lines in a diallel under stress and non-stress conditions, (ii) compare observed and predicted performance of QPM hybrids, (iii) characterize genetic diversity among the 13 QPM lines using single nucleotide polymorphism (SNP) markers and assess the relationship between genetic distance and hybrid performance, and (iv) assess diversity and population structure in 116 new QPM inbred lines as compared to eight older tropical QPM lines and 15 non-QPM lines. The GCA and SCA effects were significant for most traits under optimal conditions, indicating that both additive and non-additive genetic effects were important for inheritance of the traits. Additive genetic effects appeared to govern inheritance of most traits under optimal conditions and across environments. Non-additive genetic effects were more important for inheritance of grain yield but additive effects controlled most agronomic traits under drought stress conditions. Inbred lines CKL08056, CKL07292, and CKL07001 had desirable GCA effects for grain yield across drought stress and non-stress conditions. Prediction efficiency for grain yield was highest under optimal conditions. The classification of 139 inbred lines with 95 SNPs generated six clusters, four of which contained 10 or fewer lines, and 16 lines of mixed co-ancestry. There was good agreement between Neighbor Joining dendrogram and Structure classification. The QPM lines used in the diallel were nearly uniformly spread throughout the dendrogram. There was no relationship between genetic distance and grain yield in either the optimal or stressed environments in this study. The genetic diversity in mid-altitude maize germplasm is ample, and the addition of the QPM germplasm did not increase it measurably.

## Introduction

Maize (*Zea mays L*.) is a major staple crop of sub-Saharan Africa, where maize production was 70.6 million metric tons in 2013 and 57% (40.8 million metric tons) of which was produced in eastern and southern Africa (ESA) (FAOSTAT 2013). Most of this maize is produced under rain-fed conditions by smallholder farmers, with average yield of about 1.5 tons ha^–1^ (FAOSTAT 2013). Many maize farmers are found in the mid-altitude tropical agroecologies where a number of abiotic and biotic production constraints contribute to the low yields. Drought and low soil fertility (mainly low soil nitrogen) are the abiotic constraints most limiting maize production. Drought frequency and severity differ throughout the region, and affect maize from crop establishment through grain filling. However, drought is most devastating when it occurs at flowering, leading to delayed silking, female sterility, reduction in pollen and reduction in kernel number per plant, all resulting in reduced grain yield (Moss and Downey 1971; Hall et al. 1982). Moisture stress has been reported to reduce the rate of photosynthesis, translocation and nutrient uptake (Laude 1971; Schussler and Westgate 1991; Dwyer et al. 1992).

Loss due to drought in lowland tropical environments averages 17% (Edmeades et al. 1992) and can reach 60% in severely drought affected regions (Rosen and Scott 1992). Campos et al. (2004) reported 45–60% yield losses when drought occurred at silk emergence. Incidences of drought are expected to increase in the future due to climate change (Williams and Funk 2011), with further projected drought related production losses of 40% with each 1 °C increase in temperature (Lobell et al. 2011). Low soil nitrogen is also an important abiotic factor affecting maize production in some tropical regions. Soils in the tropics have been mined of nutrients for years without replenishment (Smaling et al. 1997), yet nitrogen status in the soil can easily be adjusted through controlled fertilizer applications. Low nitrogen increases the anthesis-silking interval (Jacobs and Pearson 1991), enhances kernel abortion (Pearson and Jacobs 1987) and reduces final grain number (Lemcoff and Loomis 1986).

In sub-Saharan Africa, maize is an important source of carbohydrates and proteins, accounting for 17–60% of people’s total daily protein supply (Krivanek et al. 2007). Such dependence on maize as the primary or only protein source can lead to protein-energy malnutrition because of its inherent deficiency in two essential amino acids, lysine and tryptophan (Nelson 1969; WHO 2000; Bright and Shwery 1983). Efforts to improve protein quality in maize led to the discovery of *opaque-2* mutants, which have increased lysine and tryptophan content (Mertz et al. 1964). The original *opaque-2* allele was associated with reduction in yield and poor kernel characteristics (Salamini et al. 1970). The *opaque-2* mutation was incorporated into maize germplasm with improved agronomic and kernel traits leading to the release of quality protein maize (QPM), which has double the amount of lysine and tryptophan compared to normal endosperm maize (Vasal et al. 1980, 1993a, b; Bressani 1991; Bjarnason and Vasal 1992; Pixley and Bjarnason 1993).

Diverse QPM genotypes adapted to sub-Saharan Africa have now been developed (Hohls et al. 1996; CIMMYT 2005; Krivanek et al. 2007; Badu-Apraku and Lum 2010; Musila et al. 2010) and their nutritional benefits for children have been documented (Akalu et al. 2010). Two approaches have been used to develop QPM germplasm adapted to ESA. In the first, inbred lines were extracted from tropical/subtropical QPM germplasm selected under ESA conditions. In the second, stress tolerant germplasm adapted to the mid-altitude of sub-Saharan Africa was converted to QPM using tropical/subtropical donor germplasm (CIMMYT 2005). The resulting genotypes were evaluated under managed stress conditions (drought and low soil nitrogen) to identify QPM genotypes tolerant to these two major stresses while maintaining protein quality. Predominantly additive (Musila et al. 2010), equal additive and non-additive (Wegary et al. 2014), and predominantly non-additive (Machida et al. 2010) effects have been reported to influence grain yield and other traits of mid-altitude tropical QPM lines developed through different methods. Continued breeding efforts have led to new stress tolerant QPM inbred lines adapted to Eastern Africa. Successful utilization of these new inbreds for hybrid development and formation of new breeding populations requires knowledge of their combining ability, which is used to elucidate the type of gene action involved in controlling quantitative characters and assist breeders to select suitable parents (Hallauer and Miranda 1988).

Breeding programs in ESA that are incorporating QPM alleles from lowland tropical/subtropical maize into adapted maize have concurrently increased the diversity in mid-altitude maize germplasm. Assessment of the new diversity can guide sound strategies for hybrid development and other uses of the germplasm. Molecular markers have been used to investigate genetic diversity and suggest heterotic groupings, hybrid testers, and possible hybrid combinations (Warburton et al. 2002; Duarte et al. 2004; Lu et al. 2009; Wen et al. 2011; Semagn et al. 2012a; Wegary et al. 2013; Akinwale et al. 2014; Badu-Apraku et al. 2015). Markers could thus be used to ascertain if an increase in diversity levels in mid-altitude maize germplasm can be measured following introduction of QPM germplasm from tropical breeding programs. In the light of new diversity coming into the African mid-altitude maize breeding pool from lowland tropical QPM breeding programs, the objectives of this study were to (i) estimate the general and specific combining abilities of 13 QPM inbred lines under stress and non-stress conditions, (ii) compare observed and predicted performance of QPM hybrids under stress and non-stress conditions, (iii) characterize genetic diversity among the 13 QPM inbred lines using single nucleotide polymorphism (SNP) markers and assess relationship between genetic distance and hybrid performance (iv) assess diversity and population structure in 116 new QPM inbred lines as compared to older tropical QPM lines and non-QPM lines from both mid-altitude and tropical breeding pools.

## Materials and methods

### Development of QPM inbred lines and hybrid formation

Thirteen QPM inbred lines developed in a CIMMYT program to introgress QPM alleles from tropical/subtropical maize from Mexico into mid-altitude adapted maize germplasm in Kenya were used to estimate combining ability parameters in this study (Table 1). Five QPM lines (entries 1–3, 9, and 11) were developed from conversion of stress tolerant normal endosperm inbred lines widely used in maize breeding programs in mid-altitude areas of ESA to QPM using inbreds CML144 and CML159, and population POOL15QPM-SR as QPM allele donors (Table 1). Six QPM lines (entries 4–8, and 10) were developed from conversion of three mid-altitude normal endosperm maize OPVs (LLSYNTH1, SYNTHSR, and ECA-MOROSR) to QPM using POOL15QPM-SR as the QPM allele donor. During development of lines from the conversion program, a single backcross to the OPV QPM donor was made to increase the frequency of modifiers for the *opaque-2* gene. Two lines (entries 12 and 13) were extracted from QPM population POOL15QPM-SR. During inbred line development, selection of kernels to advance to the next generation was done on the light table (Vivek et al. 2008). The 13 entries were inbred to the S_5_–S_7_ level and selected based on topcross performance, protein quality, and good kernel modification. Tryptophan content in the endosperm at the S2 and S4 generations was analyzed at the CIMMYT Cereal Quality Laboratory in Mexico. We only measured tryptophan content because values of tryptophan and lysine are highly correlated, and normally the value of lysine is four times the value of tryptophan (Vivek et al. 2008). The 13 entries were planted in a nursery in October 2008 at the KARI Kiboko Research Center in Kenya to make crosses in a diallel mating design. Seeds from reciprocal crosses were bulked to form a set of 78 F_1_ diallel hybrids.

**Table 1:** List of 13 quality protein maize inbred lines, their pedigrees, tryptophan content and quality index

Inbred line	Code	Pedigree	Non-QPM parent	Tryptophan (%)
1	CKL08056	((CML216/CML144//CML159)//POOL15QPMSR)-B-32-B-3-B	CML216	0.063
2	CKL07292	((CML312/CML144//CML159)//POOL15QPMSR)-B-16-B-B-B	CML312	0.078
3	CKL07361	((CML390/CML144//CML159)//POOL15QPMSR)-B-28-B-B-B	CML390	0.061
4	CKL07298	((LLSYNTH1/PL15QPMC7SRC1F2)//POOL15QPMSR)-B-17-B-B-B	LLSYNTH	0.077
5	CKL08051	((ECA-MOROSR(BC1)F2-6-ECAVEE6/PL15QPMC7SRC1F2)//POOL15QPMSR)-B-97-B-B-B	ECAMOROSR	0.076
6	CKL08052	((LLSYNTH1/PL15QPMC7SRC1F2)//POOL15QPMSR)-B-1-B-B-B	LLSYNTH1	0.076
7	CKL07303	((SYNTHSR/PL15QPMC7SRC1F2)//POOL15QPMSR)-B-82-B-B-B	SYNTHSR	0.076
8	CKL07313	((SYNTHSR/PL15QPMC7SRC1F2)//POOL15QPMSR)-B-52-B-B-B	SYNTHSR	0.074
9	CKL07325	((CML389/CML144//CML159)//POOL15QPMSR)-B-59-B-B-B	CML389	0.071
10	CKL08062	((ECA-MOROSR(BC1)F2-6-ECAVEE6/PL15QPMC7SRC1F2)//POOL15QPMSR)-B-35-B-B-B	EECAMOROSR	0.069
11	CKL07333	((CML444/CML144//CML159)//POOL15QPMSR)-B-41-B-B-B	CML444	0.069
12	CKL07004	(Pool15QPMFS462)-B-4-B-#-B-B-B	–	0.111
13	CKL07001	(Pool15QPMFS538)-B-3-B-B-B-B-B	–	0.072

### Field evaluation and stress management

The 78 F1 hybrids, one QPM single cross hybrid (CML144/CML159) commonly used as a parent of QPM three-way cross hybrids in ESA (Krivanek et al. 2007), and a commercial check were included in the trial. The field trial was evaluated in 2009 at Kiboko under optimal, managed drought stress and managed low N conditions; at Embu and Kakamega under optimal conditions; and at Namulonge under random abiotic stress (Table 2). In 2009, rainfall distribution was erratic at Namulonge and below average for this location leading to random abiotic stress. Trials under optimal conditions were rain fed except at Kiboko where we applied irrigation water. An alpha (0, 1) lattice design (Patterson and Williams 1976) with two replicates was used at each location. The experimental unit was a single 4 m row plot with inter row and intra row spacing of 0.75 and 0.20 m, respectively, for the three trials in Kiboko to give a plant density of approximately 66,667 plants ha^–1^. Two seeds were planted per hill and thinned to one plant per hill 2 weeks after emergence to give a plant density of 53,333 plants ha^–1^ at all other locations. Recommended agronomic practices including fertilizer application at each location were followed. Plots were kept weed free by hand weeding. For trials under optimal conditions and managed drought, fertilizer was applied at a rate of 27 kg N ha^–1^ and 60 kg P ha^–1^ as di-ammonium phosphate (DAP) at planting, with a second dose of 60 kg N ha^–1^ top-dressed as calcium ammonium nitrate (CAN) 4 weeks after emergence.

**Table 2:** Description of test locations used to evaluate 78 QPM hybrids and two commercial checks in 2009 and 2010

Location	Country	Longitude	Latitude	Elevation (m asl)	Mean annual rainfall (mm)	Soil types	Management	Grain yield (t ha^–1^)	
								Mean	Range
Kiboko	Kenya	36°37E	3°18’S	975	530	Sandy clay	Managed drought stress	2.9 ± 0.7	0.9–4.4
							Managed low nitrogen stress	1.8 ± 0.7	0.2–3.8
							Optimal	8.9 ± 1.9	5.3–12.2
Embu	Kenya	37°41’E	0°45^’^S	1480	1200	Clay loam	Optimal	5.7 ± 1.1	3.5–8.1
Kakamega	Kenya	34°45’E	0°16^’^N	1585	1995	Sandy loam	Optimal	8.6 ± 1.7	5.2–13.4
Namulonge	Uganda	34°04’E	0°32’N	1150	1200	Sandy clay	Random abiotic stress	1.5 ± 0.6	0.4–2.9

*m asl* meters above sea level

The trial under managed low N at Kiboko was planted in a field that had been depleted of nitrogen by growing maize continuously without N fertilizer application and removing crop biomass after each season for more than 4 years. Maize yield in a managed low nitrogen stress field is about 25–30% lower than a well-fertilized field (Bänziger et al. 2000). No N fertilizer was applied in this trial at planting and for topdressing. The trials under managed drought stress were conducted at Kiboko during the dry season (June to October) in 2009 and 2010. The June-October period at Kiboko is rain-free and thus allows for proper management of drought stress. Irrigation water was applied using sprinklers at planting and at regular intervals to establish good plant stand. Drought stress in the trials was achieved by withdrawing irrigation water 45 days after planting. Irrigation water was applied every 4 days, with each irrigation period lasting 4 hours. Total irrigation water applied from planting to the time of stopping water supply was approximately 260 mm. In these drought stress trials, average anthesis-silking interval (ASI) was calculated every 3 days after stopping irrigation to determine the need for additional water during or after flowering. The average ASI in these trials was 2.5 days so no additional irrigation water was applied based on drought stress management guidelines (Bänziger et al. 2000). At harvest, ears from plants at the two ends of each row in these trials were discarded because of less competition and access to water in the alleys between blocks in the trial.

### Data collection

Data were recorded for the following traits: days to silking (SD, number of days from planting to when 50% of the plants had extruded silks), days to anthesis (AD, number of days from planting when 50% of the plants shed pollen), ASI (the difference between SD and AD), plant height (PH, distance in centimeters from the base of the plant to the height of the first tassel branch), number of ears per plant (EPP, determined by dividing the total number of ears per plot by the number of plants harvested), husk cover (HC, number of ears with open tips expressed as a percentage of number of plants harvested), and ear aspect (on a scale of 1–5 where 1 = nice and uniform cobs with the preferred texture in the area; 5 = cobs with undesirable texture in the area). Under managed drought stress, leaf senescence was scored during grain filling on a scale of 1–10 by estimating the percentage of dead leaf area and dividing it by 10; where 1 = 10% dead leaf area, 10 = 100% dead leaf area (Bänziger et al. 2000). Under all conditions, plots were hand harvested and ear weight (in trials under optimal conditions) or grain weight (in drought and low N stress trials) and moisture content recorded. Ear weight or grain weight was used to calculate grain yield expressed in tons ha^–1^ adjusted to 12.5% moisture and 80% shelling percentage.

### SNP genotyping

For marker classification work, two new data sets were generated and data from a third set of previously genotyped lines was also analyzed. One set contained the 13 QPM lines (Table 1), and the other contained 140 lines from the CIMMYT global maize program, including mid-altitude lines developed in Kenya and tropical and sub-tropical lines (including some older QPM lines) developed in Mexico (presented in Supplemental Table 1). Twelve of the thirteen QPM lines from the first data set were also present in the second data set. Ten seeds from each of the 142 inbred lines were planted in plastic seed trays at the Biosciences for eastern and central Africa (BecA) hub in Nairobi, Kenya. Leaves were collected for DNA extraction at the 3–4 leaf stage, and extraction was done using the CIMMYT protocol (CIMMYT 2005) modified as described by Semagn et al. (2012b). Normalized DNA on 96-well plates was sent to the Cornell University Life Sciences Core Laboratories Center for SNP genotyping. DNA for one entry (number 10, or CKL08062) was of poor quality and therefore molecular data for this line was not generated, leaving only 12 lines in the first data set and 139 lines in the second data set (and 11 in common). Samples were genotyped with 95 SNP markers that were uniformly distributed across the genome (Supplemental Table 2; chosen by Semagn et al. 2012a) using an Illumina BeadStation 500 G (Illumina, San Diego, CA, USA). Further details on allele calling and error checking were as described by Semagn et al. (2012b). A third data set was used for classification where the 8 of the 13 QPM lines were compared to 92 other genotypes (Supplemental Table 3) with 2000 SNPs extracted from a GBS study published previously (Edriss et al. 2017).

### Statistical analysis

Analysis of variance for each location and across optimal environments was carried out using the PROC GLM procedure of SAS (SAS Institute 2011). Adjusted entry means were computed using the PROC MIXED procedure of SAS (SAS Institute 2011) considering entries as fixed, with replications and blocks within replicates as random effects. Days to flowering was used as a covariate in analysis of grain yield data from the drought stress trials. Entry means were separated using the least significant difference method. For diallel analysis, the two check hybrids were excluded. Combining ability analysis in a single environment was carried out following Griffing’s Method 4 Model I (Griffing 1956) of diallel analysis using DIALLEL-SAS05 program (Zhang et al. 2005) according to the following linear model:

$$X_{ijk}=\mu +r_{k}+g_{i}+g_{j}+s_{ij}+e_{ijk}$$

where *X_ijk_* is the observed performance of the cross between*i* th and *j* th parents in the *k*th replication, *η* is the population mean, *r_k_* is the replication effect, *g_i_* is the GCA effect for the ith parent, *g_i_* = the GCA effect for the jth parent, *s_ij_*= the SCA effect for the cross between *i*th and *j*th parents, and *e_ijk_* = the experimental error for the *X_ijk_* observation (Hallauer and Miranda 1988). The relative contribution of GCA (additive) and SCA (non-addtive) to the variation among hybrids for each trait was computed as percentage of the sum of squares for the crosses across different environments. The rankings of GCA effects for grain yield at each and across locations were compared using the nonparametric Spearman rank correlation coefficient. Predicted hybrid performance was performed using the additive model:

$${\text{Y}}_{\text{ij}}=\mu +g_{i}+g_{j}$$

where *y_ij_* is the predicted performance of the hybrid between inbred lines *i* and *j, μ* is the observed mean hybrid performance, and *g_i_* and *g_i_* are the GCA effects of inbred lines *i* and *j*, respectively (Charcosset et al. 1998).

### Population and structure analysis

Analysis of structure and relationships within the two data sets (139 inbred lines including 116 new QPM lines, 12 of which were used in the diallel, eight old QPM lines, 15 non-QPM lines; and 100 inbred lines including 8 of the new QPM lines and 18 non-QPM CMLs) in this study was done using both Structure (Pritchard et al. 2000) and PowerMarker (Liu and Muse 2005). The Structure analysis was run setting the number of clusters, K, from 2 to 12, to find the best fit, according to the LnP(D) statistic, by maximizing variation between clusters while minimizing it within, and by finding the classification with the fewest number of mixed individuals. K was thus set to be 6, and Structure was then run with 500,000 after 50,000 runs as a burn in, to find the best clustering of the individuals. This classification was compared to the Neighbor Joining (NJ) method calculated from Shared Allele genetic distance matrix of all individuals using PowerMarker (Liu and Muse 2005). The two methods use very different calculations and thus generate different classifications, but both should agree when lines are closely related, and when clusters are robust. The Shared Allele genetic distance matrix was also used to find distances between the 12 inbreds grown in the diallel (for which there was good SNP data) for the correlation between genetic distance and hybrid performance. A regression was run comparing genetic distance and hybrid performance under the different management conditions in Excel.

## Results

### Analysis of variance and combining ability across various management conditions

The combined analysis of variance under optimal conditions showed significant environment, genotype, F^1^ hybrid, genotype × environment, and F^1^ hybrid × environment interaction effects for all recorded traits except genotype × environment interaction and F^1^ hybrid × environment interaction for some traits (Table 3). Combined ANOVA under managed drought stress showed that environment, genotype, and F^1^ hybrid were significant for all traits recorded. Under managed low N stress genotype and F^1^ hybrids were significant for all traits recorded while Table 3 Mean squares from combined ANOVA for grain yield and agronomic traits across optimal, managed drought and all environments in 2009 and 2010 under random abiotic stress genotype and F^1^ hybrids were significant for four traits (Supplemental Table 4). Across all conditions environment, genotype, F^1^ hybrid, genotype × environment and F^1^ hybrid × environment interaction effects were significant for all traits recorded (Table 3). General combining ability and SCA were significant for all traits except SD, PH and HC across optimal conditions (Table 3). Across managed drought stress, GCA was significant for all traits. Across all conditions, GCA and SCA were significant for all traits. The interaction of both GCA and SCA with the environment (GCA × E and SCA × E) was significant for two traits under optimal conditions (Table 3). Assessment of the relative contribution of GCA and SCA sum of squares to hybrid variation showed that GCA accounted for the majority of the variation (58–80%) for most traits under optimal, managed drought stress, and across all environments (Table 3). The contribution of GCA sum of squares among hybrids for GY was similar under optimal and low N stress but different for managed drought stress conditions.

**Table 3:** Mean squares from combined ANOVA for grain yield and agronomic traits across optimal, managed drought and all environments in 2009 and 2010

Optimal conditions									
	df	GY (t ha^–1^)	AD (days)	SD (days)	ASI (days)	EPP (no.)	PH (cm)	HC (%)	EA (1–5)
Environment (E)	2	484.72***	7620.98***	5707.06***	159.01***	1.20***	24813.72***	1680.12***	6.74***
Rep (E)	3	23.68***	11.34***	9.49**	1.65	0.03*	2594.12***	238.86*	6.98***
Genotype	79	5.72***	23.78***	24.62***	4.83***	0.02***	884.30***	585.55***	0.60***
F1 Hybrids	77	5.15***	20.11***	21.46***	4.80***	0.02***	846.69***	593.34***	0.57***
GCA	12	22.43***	103.16***	109.45	17.81***	0.04***	4110.23	1704.00	2.60***
SCA	65	1.96*	4.77***	5.21	2.40**	0.01**	244.19	388.30	0.19*
Genotype 9 E	158	2.79***	2.35*	2.78**	1.63	0.02***	209.94	156.90***	0.26***
F1 Hybrids 9 E	154	2.71***	2.11	2.49*	1.67	0.02***	184.64	160.46***	0.26***
GCA 9 E	24	5.30	2.31***	8.73	2.72*	0.03***	261.60	367.42	0.72***
SCA 9 E	130	2.23	0.95	1.34	1.47	0.01*	170.43	122.25	0.17*
Error	237	1.40	1.80	1.87	1.47	0.01	191.59	70.21	0.14
%GCA SS		62	80	80	58	34	76	45	72
%SCA SS		38	20	20	42	66	24	55	28

Managed drought stress									
	df	GY (t ha^–1^)	AD (days)	SD (days)	ASI (days)	EPP (no.)	HC (%)	EA (1–5)	SEN (1–9)
Environment (E)	1	62.22***	1344.80***	790.65***	73.15***	1.94***	565.52**	23.65***	1.44***
Rep (E)	2	9.95***	6.53***	30.92***	9.80*	0.34***	54.61	7.66***	0.07***
Genotype	79	1.38***	17.26***	30.62***	11.19***	0.06***	65.51	0.75***	0.02***
F1 Hybrids	77	1.28**	12.15***	22.07***	10.99***	0.05***	66.35	0.68***	1.94***
GCA	12	3.88***	63.24***	108.26***	51.08***	0.16***	128.99*	2.52***	7.91***
SCA	65	0.80	2.72***	6.16***	3.59*	0.03	54.78	0.34	0.84
Genotype 9 E	79	0.67	1.88***	5.93**	5.34**	0.02	62.66	0.32	0.01
F1 Hybrids 9 E	77	0.68	1.83***	5.21***	5.08***	0.03	63.92	0.33	0.8
GCA 9 E	12	1.35*	5.41***	14.15***	16.35***	0.03	83.51	0.57*	0.79
SCA 9 E	65	0.56	1.17*	3.56	3.00	0.03	60.30	0.28	0.80
Error	158	0.73	0.75	3.44	2.97	0.03	60.58	0.25	0.01
%GCA SS		47	81	76	72	50	30	58	63
%SCA SS		58	19	24	28	50	70	42	37

Across environments										
	df	GY (t ha^–1^)	AD (days)	SD (days)	ASI (days)	EPP (no.)	HC (%)	EA (1–5)	df	PH (cm)
Environment (E)	6	1617.05***	4831.37***	3192.23***	627.02***	2.54***	1907.84***	33.47***	5	341253.94**
Rep (E)	7	15.07***	20.88***	103.99***	13.43**	0.12***	150.51*	6.59***	6	3958.53***
Genotype	79	4.07***	16.05***	85.07***	23.37***	0.05**	559.71***	0.95***	79	1725.08***
F1 Hybrids	77	3.87***	41.47***	66.44***	23.53***	0.05***	565.44***	0.90***	77	1723.20***
GCA	12	16.55***	211.80***	324.03***	112.63***	0.16***	1715.30***	3.35***	12	8824.47***
SCA	65	1.52***	10.03***	20.06***	6.50***	0.03*	353.16***	0.45**	65	412.19***
Genotype 9 E	474	1.73***	1.33***	10.85***	6.91***	0.03***	157.20***	0.41***	395	249.07**
F1 Hybrids 9 E	462	1.62***	3.26**	10.14***	6.76***	0.03***	160.21***	0.39***	385	228.46*
GCA 9 E	72	4.04***	5.20***	4.62	3.26	0.06***	355.93***	0.99***	72	302.32**
SCA 9 E	390	1.17**	2.90*	4.44	2.25	0.03**	124.08***	0.28	325	169.72
Error	553	0.90	2.59	6.54	4.00	0.02	76.32	0.27	474	199.15
%GCA SS		67	80	75	76	50	47	58		80
%SCA SS		33	20	25	24	50	53	42		20

*AD* days to anthesis, *ASI* anthesis-silking interval, *EA* ear aspect, *EPP* ears per plant, *GY* grain yield, *HC* husk cover, *PH* plant height, *SD* days to silking, *SEN* leaf senescence

* Significant at the P < 0.05 level of probability

** Significant at the P < 0.01 level of probability

*** Significant at the P < 0.001 level of probability

### GCA and SCA effects

The GCA effects of the QPM lines for grain yield at each location, across optimal and managed drought, and under low N stress are presented in Table 4. The GCA effects for GY across optimal conditions were significant and positive for inbred lines P1 (CKL08056), P2 (CKL07292) and P13 (CKL07001). Across managed drought stress inbred line P1 (CKL08056) had the highest and significant GCA effect for GY (0.29 t ha^–1^). Under managed low N stress, five inbred lines showed significant positive GCA effects for GY. Three of the lines with significant positive GCA effects for GY under low N stress also had positive GCA effects under managed drought stress. The GCA effects for agronomic traits of the QPM lines are presented in Table 5. There were two inbred lines with significant positive GCA effects for EPP and four inbred lines with desirable GCA effects for reduced senescence under managed drought stress. There was a strong correlation between the ranks of GCA effects across optimal and drought conditions. The ranks of GCA effects at Kakamega were strongly associated with those at other locations except Embu. There was no significant association between the ranks of GCA effects under low N with those under any other condition (Supplemental Table 5).

**Table 4:** Estimates of general combining ability (GCA) effects for grain yield (t ha^–1^) of 13 QPM inbred lines at individual locations and across conditions

Line	Optimal conditions				Drought stress			Low N stress
	Embu	Kakamega	Kiboko	Across	Kiboko 2009	Kiboko 2010	Across	
1	0.40	1.07***	1.51***	1.00***	0.44*	0.15	0.29*	0.18
2	0.49	1.39***	0.67***	0.85***	0.49*	− 0.01	0.24	− 0.43***
3	− 0.34	− 0.98**	0.47**	− 0.28	− 0.10	− 0.47**	− 0.29*	0.43***
4	0.22	− 0.50	− 0.27	− 0.18	0.26	0.24	0.25	0.46***
5	− 0.56*	0.08	0.34	− 0.05*	0.27	− 0.06	0.11	0.44***
6	− 0.61*	0.56	− 0.648***	− 0.23	− 0.35	0.43**	0.04	0.22
7	0.08	− 0.41	− 0.50**	− 0.27	− 0.70**	− 0.50**	− 0.60***	− 0.52***
8	0.30	− 0.28	0.31	0.11	0.03	0.28	0.16	− 0.04
9	− 0.65*	− 0.59	− 0.91***	− 0.72**	− 0.04	0.02	− 0.01	− 0.47***
10	− 0.28	− 0.33	− 0.69***	− 0.43	− 0.26	− 0.33*	− 0.30*	− 0.36**
11	− 0.47	0.04	− 0.32	− 0.25	0.28	0.21	0.25	0.41**
12	0.29	− 0.77*	− 1.19***	− 0.56*	− 0.37	− 0.42**	− 0.40**	− 0.06
13	1.12***	0.71*	1.22***	1.02***	0.06	0.46**	0.26	− 0.28*
SE(g_i_)	0.28	0.31	0.18	0.25	0.21	0.15	0.15	0.12

* Significant at the *P* < 0.05 level of probability

** Significant at the *P* < 0.01 level of probability

*** Significant at the *P* < 0.001 level of probability

*SE* standard error of GCA effects

**Table 5:** Estimates of general combining ability (GCA) effects for some agronomic traits of 13 QPM inbred lines across different conditions

	Days to anthesis				Anthesis-silking interval				Plant height				SEN
	Optimal	Drought stress	Low N stress	Random stress	Optimal	Drought stress	Low N stress	Random stress	Optimal	Drought stress	Low N stress	Random stress	Drought stress
1	1.19	0.69	1.07***	1.01	0.58**	0.54*	−0.14	1.22*	−2.24	−4.71*	−4.43*	−17.07***	−0.46**
2	1.71*	1.10**	1.25***	0.97	−0.07	−0.21	0.45	0.04	6.20**	7.09**	6.57***	2.70	−0.48**
3	−0.25	−0.63	−0.52*	−0.44	0.11	0.65*	−1.37	1.68**	1.76	−1.94	−2.16	−5.02	0.52**
4	−1.69*	−1.70***	−1.48***	−1.71*	0.74***	0.54*	−0.78	1.04*	8.18***	13.22***	10.03***	7.20	0.25
5	−0.05	0.73*	0.02	−1.03	−1.03***	−2.05***	−5.46***	−2.41***	2.20	3.99	−3.27*	9.52*	0.64***
6	−2.69***	−2.58***	−2.02***	−1.99**	0.05	−0.64*	0.95	−0.28	−17.11***	−13.14***	−17.04***	−11.84*	0.26
7	0.48	0.62	−0.29	−0.03	−0.09	1.79***	2.81***	0.09	−7.31***	−11.05***	−14.91***	−12.89*	−0.26
8	0.25	0.12	−0.75**	0.74	0.37*	0.54*	0.91	0.63	6.20**	3.06	3.03	7.52	−0.16
9	−0.34	−0.36	0.12	−0.80	−0.68***	−1.17***	1.72*	−3.09***	−6.61**	−2.12	−1.04	7.84	0.17
10	−0.36	−0.20	−0.43	1.33	0.12	0.22	0.68	0.95	−6.40**	−12.12***	−7.09***	−8.39	0.29
11	−0.05	0.10	0.75**	0.06	−0.48**	−1.03***	−2.59**	−0.41	−1.84	2.20	2.55	−2.16	0.28
12	−0.13	−0.02	0.07	−0.44	0.61***	1.40***	2.36**	0.00	6.02**	5.04*	9.41***	9.20	−0.71***
13	1.93*	2.12***	2.21***	2.33**	−0.22	−0.58*	2.21***	0.54	10.96***	10.47***	18.34***	13.38**	−0.35*
SE(_g_i__)	0.75	0.38	0.24	0.75	0.18	0.27	0.24	0.53	2.15	2.43	1.82	4.88	−0.46**

	Ears per plant				Ear aspect				Husk cover			
	Optimal	Drought stress	Low N stress	Random stress	Optimal	Drought stress	Low N stress	Random stress	Optimal	Drought stress	Low N stress	Random stress
1	0.02	0.05	0.04	0.09*	− 0.30***	− 0.16	0.08	− 0.02	− 2.66*	− 0.18	− 4.60*	− 1.49
2	− 0.03*	− 0.02	− 0.09***	0.05	− 0.21***	− 0.20*	0.30**	0.07	4.78***	− 0.19	2.42	5.36*
3	− 0.01	0.01	0.09**	− 0.01	0.05	0.27**	− 0.45***	0.25	7.66***	1.47	0.65	11.90***
4	0.02	0.06*	0.03	− 0.03	0.20**	0.05	0.01	0.04	4.92***	0.95	− 0.53	− 0.68
5	0.05**	0.08**	0.12***	0.00	− 0.08	− 0.32**	− 0.29**	− 0.30	− 6.85***	− 2.66	− 6.43***	− 3.14
6	0.02	0.03	0.02	− 0.03	0.04	− 0.07	0.08	0.34	− 2.47*	− 0.33	− 0.72	− 6.22**
7	− 0.03	− 0.15***	− 0.08**	0.00	0.04	− 0.05	0.19*	− 0.05	− 3.95**	− 0.27	0.42	0.39
8	0.01	0.00	− 0.08**	0.05	0.23***	0.28**	0.19*	− 0.05	4.41***	3.64**	12.48***	2.72
9	− 0.02	0.01	− 0.09**	0.10	0.19**	− 0.15	− 0.02	− 0.41*	− 3.58**	− 1.04	0.52	3.55
10	0.00	− 0.01	− 0.05	− 0.12**	0.00	0.26**	0.17	0.13	− 1.96	− 2.03	4.70*	− 5.71*
11	− 0.04*	0.02	0.05*	− 0.02	0.13*	0.28**	− 0.17	0.29	− 6.04***	− 0.07	− 8.02***	− 1.91
12	0.00	− 0.07*	− 0.04	0.02	0.12*	0.21*	− 0.20*	− 0.18	7.30***	2.09	− 3.72*	− 6.31**
13	0.01	− 0.01	0.06*	− 0.09	− 0.41***	− 0.38***	0.12	− 0.12	− 1.57	− 1.38	2.84	1.55
SE (_g_i__)	0.16	0.03	0.03	0.04	0.06	0.10	0.12	0.19	1.27	1.66	1.81	2.25

*SEN* leaf senescence, *SE* standard error of *GCA* effects

* Significant at the *P* < 0.05 level of probability

** Significant at the *P* < 0.01 level of probability

*** Significant at the *P* < 0.001 level of probability

### Hybrid performance and prediction

Mean grain yield ranged from 6.1 to 10.2 t ha^–1^ and 1.3 to 4.2 t ha^–1^ across optimal and managed drought stress conditions, respectively (Supplemental Fig. 1). Mean performance of the top 30 QPM hybrids, SCA effects and repeatability under optimal conditions and managed drought stress is presented in Tables 6 and 7, respectively. The best two hybrids under optimal conditions significantly out yielded the QPM single cross check (CML144/CML159) by 22.6 and 19.8%, respectively. Eleven out of the top 30 QPM hybrids under optimal conditions had inbred lines P1 (CKL08056) and P13 (CKL07001) as one of their parents. The top two hybrids across managed drought stress out yielded the commercial check by 35.5 and 31.6%, respectively (Table 7). Inbred lines P1 (CKL08056) and P13 (CKL07001) were parents to the largest number of top performing hybrids under managed drought stress. Repeatability estimates ranged from 0.21 for EPP to 0.91 for AD, and 0.04 for HC to 0.90 for AD under optimal conditions and managed drought stress, respectively.

**Table 6:** Mean grain yield and agronomic traits of the top 30 F1 QPM hybrids evaluated across optimal conditions in 2009

Entry	Hybrid	GY (t ha^–1^)	AD (days)	SD (days)	ASI (days)	PH (cm)	EPP (#)	HC (%)	EA (1–5)	SCA (GY) (t ha^–1^)
1	1 × 2	10.2	66	67	1	227	1.0	16	1.9	0.63
21	2 × 11	9.9	65	66	1	232	1.0	4	2.3	1.54*
12	1 × 13	9.8	68	69	1	230	1.0	0	1.8	0.11
42	4 × 13	9.4	63	64	1	254	1.1	20	2.4	0.88
18	2 × 8	9.2	65	66	1	235	1.0	40	2.7	0.51
7	1 × 8	9.0	63	65	3	234	1.1	3	2.4	0.23
9	1 × 10	9.0	65	65	1	214	1.0	4	1.9	0.68
23	2 × 13	8.9	68	68	0	229	1.0	4	1.8	−0.63
17	2 × 7	8.9	65	66	0	236	1.0	12	2.4	0.64
78	12 × 13	8.9	65	65	1	239	1.0	11	2.1	0.71
13	2 × 3	8.7	66	66	1	230	1.0	23	2.3	0.46
77	11 × 13	8.7	66	65	0	235	1.0	1	2.3	0.13
8	1 × 9	8.7	66	65	−1	216	1.1	4	2.5	0.69
3	1 × 4	8.7	63	65	2	236	1.2	25	2.5	0.15
50	5 × 13	8.7	64	65	1	243	1.1	0	2.0	−0.02
58	7 × 8	8.6	64	66	1	219	1.1	12	2.8	1.04
68	8 × 13	8.6	66	66	0	236	1.1	15	2.5	−0.26
5	1 × 6	8.6	61	62	1	200	1.1	7	2.3	0.09
33	3 × 13	8.6	65	65	0	239	1.0	23	2.3	0.12
4	1 × 5	8.5	65	65	0	224	1.1	2	2.0	−0.14
15	2 × 5	8.5	64	63	−1	221	1.0	3	2.3	−0.04
2	1 × 3	8.4	66	67	1	225	1.0	21	2.2	0.01
63	7 × 13	8.3	66	64	−2	216	1.0	3	2.1	−0.11
14	2 × 4	8.1	64	65	1	239	1.1	6	2.4	−0.28
57	6 × 13	8.0	64	64	0	215	1.0	2	2.1	−0.45
51	6 × 7	8.0	60	61	1	201	1.0	3	2.6	0.84
75	10 × 13	7.9	65	66	1	237	1.1	2	2.3	−0.36
66	8 × 11	7.9	64	64	0	236	1.1	4	2.8	0.31
11	1 × 12	7.8	65	66	1	224	1.2	3	2.3	−0.30
43	5 × 6	7.8	60	60	−1	216	1.2	1	2.4	0.42
79	CML144/CML159	8.2	70	69	−1	246	1.0	0	2.3	
80	H513	10.7	67	69	1	241	1.1	7	1.8	
Trial mean		7.8	64	64	0	224	1.0	9	2.5	
Mean of top 30 F1 hybrids		8.7	64	65	1	228	1.1	9	2.3	
Mean of checks		9.5	69	69	0	243	1.0	4	2.0	
LSD_(0.05)_		1.4	2	2	1	16	0.1	10	0.4	0.78^†^
Repeatability		0.52	0.91	0.89	0.68	0.82	0.21	0.74	0.61	

*AD* anthesis date, *ASI* anthesis-silking interval, *EA* ear aspect, *EPP* ears per plant, *GY* grain yield, *HC* husk cover, *PH* plant height, *SCA* specific combining ability, *SD* days to silking

† Standard error of *SCA* effects

**Table 7:** Mean grain yield and agronomic traits of the top 30 F1 QPM hybrids evaluated across managed drought stress in 2009 and 2010

Entry	Hybrid	GY (t ha^–1^)	AD (days)	SD (days)	ASI (days)	EPP (no.)	EA (1–5)	SEN (1–9)	HC (%)	SCA (GY) (t ha^–1^)
21	2 × 11	4.2	68	68	1	1.0	3.3	8	0	0.93*
1	1 × 2	4.1	67	68	2	1.0	2.8	8	5	0.78
15	2 × 5	4.1	67	66	−1	1.0	2.0	8	2	0.92*
66	8 × 11	4.0	66	66	1	0.9	3.9	9	1	0.81
40	4 × 11	3.9	65	67	3	1.0	3.4	9	3	0.54
68	8 × 13	3.8	68	71	3	0.9	2.9	8	5	0.52
33	3 × 13	3.7	68	69	1	1.1	3.1	8	6	0.91*
72	9 × 13	3.5	69	68	−1	0.9	3.0	8	0	0.43
7	1 × 8	3.4	67	70	3	1.0	3.5	8	3	0.14
45	5 × 8	3.4	67	67	0	1.0	2.9	9	4	0.33
8	1 × 9	3.4	67	68	1	1.0	2.8	9	6	0.25
23	2 × 13	3.3	70	71	1	0.8	2.8	8	1	−0.01
5	1 × 6	3.3	64	67	3	0.9	3.1	9	3	0.11
38	4 × 9	3.3	65	67	2	0.9	3.1	8	5	0.20
4	1 × 5	3.2	67	68	1	1.0	2.8	8	8	0.02
28	3 × 8	3.2	65	68	3	1.0	3.3	8	16	0.49
3	1 × 4	3.2	66	68	2	0.9	3.4	8	3	−0.20
16	2 × 6	3.2	65	66	1	0.9	3.0	8	2	0.06
29	3 × 9	3.2	65	67	2	0.9	2.9	9	1	0.63
42	4 × 13	3.2	67	69	2	0.9	3.3	9	4	−0.17
76	11 × 12	3.2	66	68	2	0.9	3.9	7	6	0.07
57	6 × 13	3.1	65	66	1	0.9	2.6	8	0	0.01
35	4 × 6	3.1	60	63	3	1.0	3.3	9	5	0.00
41	4 × 12	3.1	64	67	4	1.0	3.1	8	6	0.44
78	12 × 13	3.1	67	70	3	0.8	2.8	7	8	0.42
48	5 × 11	3.1	67	67	0	1.0	3.1	9	1	−0.11
39	4 × 10	3.0	63	66	3	0.9	3.1	10	0	0.26
9	1 × 10	3.0	67	71	4	0.8	3.3	8	4	0.14
34	4 × 5	3.0	65	66	1	0.9	3.3	9	4	−0.22
53	6 × 9	3.0	64	65	1	0.8	3.3	8	4	0.11
79	CML144/CML159	1.2	76	81	5	0.5	4.5	8	1	
80	H513	3.1	70	73	3	0.7	2.8	7	1	
Trial mean		2.8	66	68	2	0.8	3.3	8	4	
Mean of top 30 F_1_ hybrids		3.4	66	67	2	0.9	3.1	8	4	
Mean of checks		2.2	73	77	4	0.6	3.6	8	1	
LSD_(0.05)_		1.2	1	3	2	0.2	0.7	1	11	0.46^a^
Repeatability		0.53	0.90	0.39	0.52	0.56	0.27	0.19	0.04	

*AD* days to anthesis, *ASI* anthesis silking interval, *EA* ear aspect, *EPP* ears per plant, *GY* grain yield, *HC* husk cover, *SCA* specific combining ability, *SD* days to silking, *SEN* leaf senescence

a Standard error of *SCA* effects

**Fig. 1:**
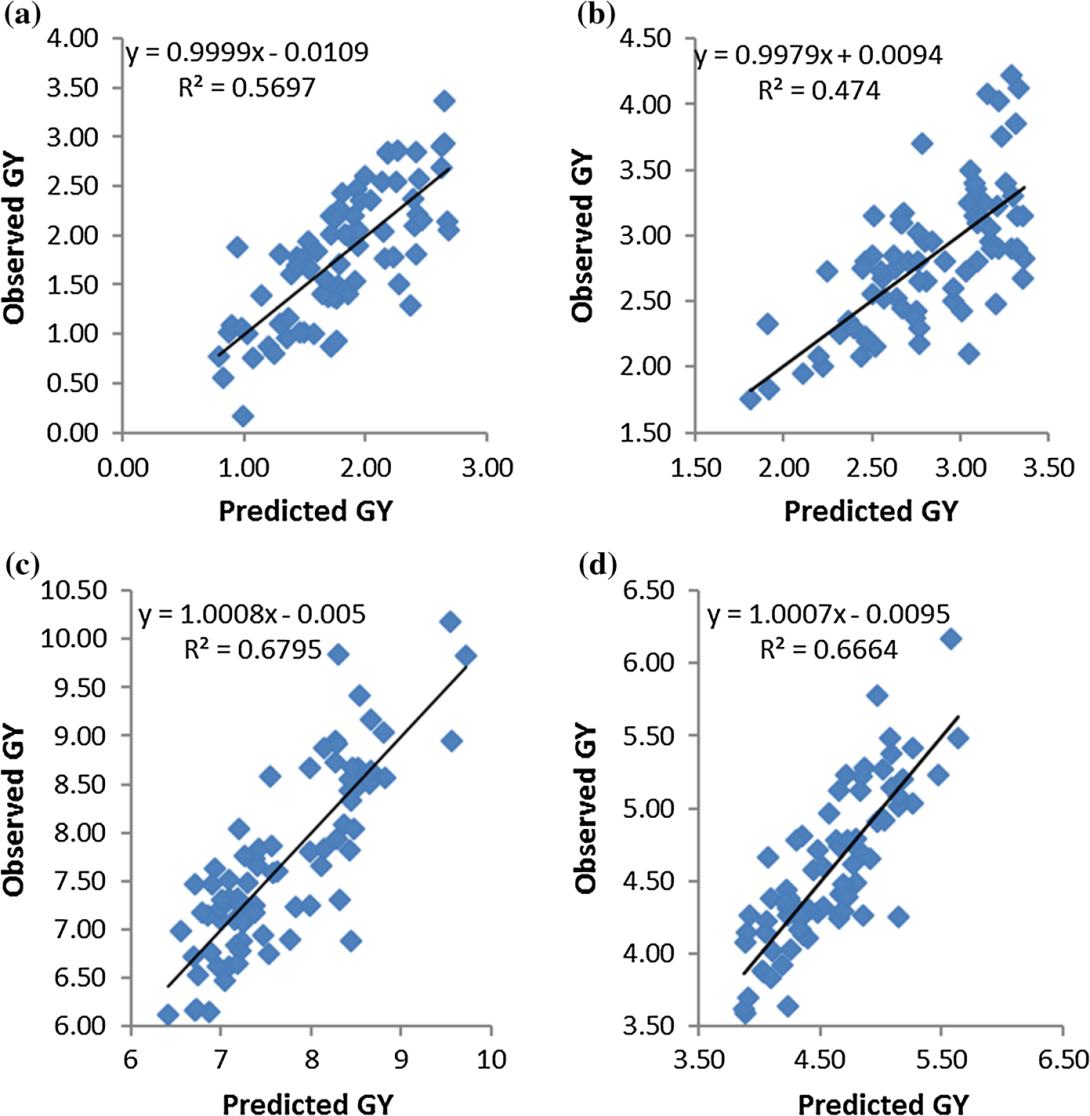
Relationship between predicted and observed grain yield (t ha^–1^): (a) under managed low nitrogen stress at Kiboko, (b) across managed drought stress at Kiboko, (c) across optimal conditions, and (d) across all environments

Predicted hybrid performance was highly correlated with observed F_1_ hybrid performance for all locations except in Namulonge where the hybrids experienced random abiotic stress (data not shown). Prediction efficiency for GY ranged from 0.47 to 0.68 under different management conditions (Fig. 1). Of the top 20 crosses predicted to have the highest GY, 75, 65, 50 and 60% were among the top 20 hybrids in terms of performance across all conditions, optimal, managed drought stress and under low nitrogen, respectively (data not shown). The top 20 hybrids predicted to have the best performance across optimal and all conditions, and 75% of the top 20 across managed drought stress had inbred lines P1 (CKL08056), P2 (CKL07292), or P13 (CKL07001) as one of the parents (data not shown).

### Genetic distances and population structure

**Fig. 2:**
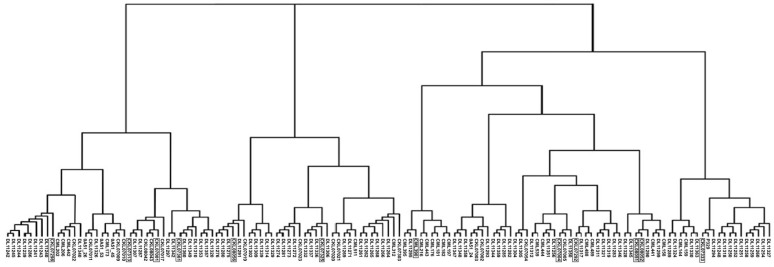
Neighbor Joining dendrogram of 139 inbred maize lines classified with 95 SNP markers calculated from the Shared Allele genetic distance matrix of all individuals

The classification of 139 inbred lines with 95 SNPs by Structure led to six clusters, four of which contained 10 or fewer lines, and 16 lines of mixed co-ancestry (Supplemental Table 6). The Neighbor Joining dendrogram shown in Fig. 2 showed good agreement with the Structure classification, although there were eight clusters in the NJ dendrogram. The 12 new QPM lines fall into three of the six Structure clusters (including the largest two) and six of the eight dendrogram clusters, and are nearly uniformly spread throughout the dendrogram (Fig. 2). Mid-altitude adapted lines in this sample are much more diverse than the tropical lines, but this is a function of the much larger mid-altitude sample size compared to tropical lines included in this sample. Because of this, tropical lines occur mainly in three of the eight dendrogram clusters and two of the six Structure clusters (albeit the largest ones, Supplemental Table 6).

The classification of 100 inbred lines with 2000 SNPs is not directly comparable to the classification using 139 lines and 95 SNPs because many of the lines differed between the two studies. However, the general conclusions regarding diversity levels reached from the classification of the 95 SNPs is confirmed with the classification of 2000 SNPs. The QPM lines fell into six dendrogram clusters via NJ, while the majority of the tropical lines fell into only two of the six dendrogram clusters (data not shown), indicating less diversity due to a smaller sample of tropical lines (24) compared to the total (100). The 8 new QPM lines genotyped with the 2000 SNPs could be directly compared to 8 older CIMMYT QPM lines included in the same study, some of which have been used as QPM donors in African breeding programs. Four clusters were detected by Structure (Supplemental Table 3). The older QPM lines fell into Structure clusters 1, 2, and 3, and all 8 new QPM lines used in the diallel belong to Structure cluster 3. The slope of the regression line between Shared Allele genetic distance and grain yield was 0.03 for the well-watered experiment and 0.03 for the water stressed experiment.

## Discussion

Breeding efforts to develop QPM germplasm for mid-altitude ESA initially relied on germplasm from the tropical lowlands of Mexico but later incorporation of the QPM trait in stress tolerant maize adapted to ESA was initiated. Availability and mode of inheritance of genetic variation is important in any breeding program as it has implications on the progress that can be made from selection. There was large genetic variability among this set of QPM lines which suggested that progress from selection and genetic gains can be made (Falconer and Mackay 1996) in a breeding program utilizing this QPM germplasm. The QPM inbred lines used in this study were developed from conversion of a variety of mid-altitude normal endosperm inbred lines and OPVs to QPM and this explained the observed large genetic variability for various traits. This result is consistent with reports of genetic variability in tropical and temperate QPM germplasm (Spaner et al. 1992; Vasal et al. 1993a; Pixley and Bjarnason 1993; Bhatnagar et al. 2004; Musila et al. 2010; Badu-Apraku and Lum 2010; Badu-Apraku et al. 2016) and in *opaque-2* types (Motto et al. 1978; Wessel-Beaver et al. 1985).

The highly significant F_1_ hybrid x environment interaction for all traits across all conditions, including optimal, suggested that the QPM genotypes evaluated in this study responded differently across the testing environments, which indicated the need for wide testing of QPM genotypes across varying environmental conditions to identify the best performing and stable hybrids that can be released for commercial production. The QPM hybrids showed consistent performance under managed drought stress across the 2 years. The lack of year-to-year variation for GY among these hybrids under managed drought stress may be explained by the convention of planting such trials at Kiboko in June of every year to coincide with the rain-free period. Some of the inbred lines used in this study were partially derived from OPVs, and this may have led to the lack of significant SCA effects under managed drought and low N stress since separation of the lines into heterotic groups was not done beforehand (Pixley and Bjarnason 1993). Repeatability estimates were moderate to high for most of the traits under different conditions due to the large genetic variation among this set of QPM germplasm. Estimates of repeatability for GY in this QPM germplasm were slightly lower than in a previous QPM study (Betrán et al. 2006).

In this study, there was larger contribution of GCA sum of squares compared to SCA sum of squares for GY and most of the agronomic traits under optimal conditions and across environments. This result suggested that inheritance of GY and some agronomic traits in these QPM inbred lines is largely controlled through additive genetic effects under optimal conditions. This result is consistent with findings in several studies with QPM germplasm (Pixley and Bjarnason 1993; Vasal et al. 1993a, b; Musila et al. 2010; Wegary et al. 2014). In contrast, studies by Bhatnagar et al. (2004) and Machida et al. (2010) showed that non-additive genetic effects were more important than additive genetic effects for control of GY in QPM germplasm. With preponderance of additive gene action for these traits and large genetic variability, a recurrent selection method like half-sib family recurrent selection that emphasizes GCA can be used effectively to improve these traits in this germplasm. The SCA sum of squares were of greater magnitude than GCA sum of squares for GY under drought stress which suggested that non-additive genetic effects were more important for control of GY in this set of QPM germplasm under drought stress. This finding is consistent with studies by Makumbi et al. (2011) and Badu-Apraku et al. (2016) but contrary to other studies (Betrán et al. 2003a; Wegary et al. 2014). The difference in the results of this study and other studies could be attributed to the differences in the type of germplasm used.

In the present study, there was significant GCA × E interaction mean squares for a large number of traits which indicated that there was variation in the GCA effects of the lines under different environments used in this study. This result is consistent with findings in several studies (Betrán et al. 2003c; Makumbi et al. 2011; Badu-Apraku et al. 2013). Extensive testing of inbred lines in multiple stress environments over seasons and/or years is therefore necessary to identify the best lines with consistent performance across the different environments for hybrid development. Three QPM inbred lines (CKL08056, CKL07292, and CKL07001) with positive significant GCA effects for GY under optimal and drought stress conditions were identified. This suggested that these three inbred lines have the potential for use in QPM breeding programs that target development of hybrids suitable for both conditions. Inbred line CKL07001 was also reported to have consistently good GCA effects under various conditions in a previous study (Musila et al. 2010), further supporting the potential of this line as a good donor parent for yield alleles under multiple environments. Lines that show consistent positive GCA effects for GY across a range of environments are good candidates for use in a QPM inbred line recycling program. In this study, inbred lines with positive GCA effects for GY under optimal conditions were of late maturity but some inbred lines with desirable positive GCA effects for GY under drought and low nitrogen stress and early maturity were identified. This implied that some of these inbred lines have the potential to be used in developing QPM hybrids combining early maturity and tolerance to some of the major abiotic stresses of maize. Some inbred lines like CKL08056 and CKL08051 that showed consistently positive GCA effects for EPP across all conditions have good potential for breeding for low nitrogen and drought stress conditions in which increased number of EPP are important for higher yield.

The yield of some of the experimental single cross hybrids was significantly better than that of the widely used parental QPM single cross hybrid CML144/CML159 under optimal conditions and under stress conditions, which suggests that they have the potential to serve as parents of productive QPM hybrids. This is especially important because most of the QPM hybrids in Eastern Africa are marketed by small-scale seed companies that produce seed under irrigated conditions and these single-crosses would be useful in such environments. Prediction of single-cross performance is an important component of hybrid breeding. In the current study, predicted hybrid performance for GY was highly correlated with observed hybrid performance under most of the conditions. These results are consistent with the findings of Welcker et al. (2005) in maize under acid soils and Makumbi et al. (2011) in maize under low N, drought and optimal conditions.

The prediction efficiencies were relatively high despite the relatedness between the lines used in this study. Prediction efficiency increased when hybrids between related lines were excluded from the prediction (Charcosset et al. 1998). A strong correlation between actual and predicted performance indicates that GCA contributes a large proportion to the differences between crosses compared to SCA (Zuber et al. 1973; Charcosset et al. 1993) and may also be due to increased precision of GCA estimates (Charcosset et al. 1998). For hybrid prediction to be beneficial in QPM breeding programs, further studies that incorporate molecular marker information and best linear unbiased prediction (BLUP) in the prediction models will be needed to verify the usefulness of hybrid prediction methods for QPM genotypes (Charcosset et al. 1998; Smith et al. 1999; Schrag et al. 2009, 2010; Kadam et al. 2016).

There was no relationship between genetic distance and grain yield in either the optimal or water stressed environments in this study. This has been observed in many previous studies (Godshalk et al. 1990; Melchinger et al. 1990; Reif et al. 2003; Dhliwayo et al. 2009; Menkir et al. 2010; Badu-Apraku et al. 2013) where the predictive ability of markers on performance is generally good only with very closely related lines, where heterosis is likely to be quite low in general. These results are in contrast to Betrán et al. (2003b) and Wegary et al. (2013) who reported significant correlation between genetic distance and grain yield. The poor correlation between GD and hybrid performance could be attributed to lack of linkage between the SNP markers used to estimate GD and quantitative trait loci for grain yield (Bernardo 1992). The markers will probably only be useful to rule out crosses between closely related lines for which pedigree data is missing, and this would be unusual. It is recommended that maize breeders and seed producers continue to rely on reported field performance of hybrids, including the data presented in the current study, to determine which hybrids to make.

In the sample of 139 inbred lines analyzed with 95 SNP markers, a clear classification was noted with both Structure and with NJ analyses. It is apparent that the genetic diversity in mid-altitude maize germplasm is ample, and likely has been since the inception of the breeding program. Due to the high levels of initial diversity, the addition of the QPM breeding material did not increase it measurably. While Structure tended to cluster the tropical lines separately from the mid-altitude lines, in the NJ dendrogram it can be seen that these tropical clusters fall within and very related to larger clusters containing the new QPM lines included in the current study. Thus, the current marker classification is most likely indicating that much of the diversity that could have been contributed from tropical material was already present in the mid-altitude breeding pool due to past breeding methodology and shared germplasm between CIMMYT breeding programs. The addition of these lines did not create highly divergent clusters of new QPM lines, indicating that the new lines apparently will not overly perturb the genetic structure of mid-altitude maize germplasm, suggesting an easier incorporation of these QPM sources into the mid-altitude maize breeding program.

## Conclusions

This study revealed large genetic variation among new QPM inbred lines developed in a conversion program. This large genetic variation will be useful in making progress from selection when developing new QPM germplasm. A number of inbred lines suitable for use in QPM breeding programs for line and hybrid development because of good GCA effects for several agronomic traits for both stressed and non-stress conditions were identified. The incorporation of the QPM trait did not appear to increase diversity among the highly diverse mid-altitude maize germplasm beyond that already present.

## Abbreviations

ASI: Anthesis-silking interval
CML: CIMMYT maize line
EPP: Ears per plant
ESA: Eastern and Southern Africa
GCA: General combining ability
GY: Grain yield
KALRO: Kenya Agricultural and Livestock Research Organization
OPV: Open-pollinated variety
QPM: Quality protein maize
SCA: Specific combining ability

## Notes

**Open Access** This article is distributed under the terms of the Creative Commons Attribution 4.0 International License (http://creativecommons.org/licenses/by/4.0/), which permits unrestricted use, distribution, and reproduction in any medium, provided you give appropriate credit to the original author(s) and the source, provide a link to the Creative Commons license, and indicate if changes were made.
