# Supplementary material for: Genetic analysis of tropical quality protein maize (Zea mays L.) germplasm
Source: Euphytica. 2017 Nov 3;213:261. doi: 10.1007/s10681-017-2048-4 (PMC7734200; doi:10.1007/s10681-017-2048-4)
Supplement: Supplementary file 4 [file EUP-213-261-s004.docx]

Supplemental Table 4. Mean squares from ANOVA for grain yield and agronomic traits under low nitrogen and random abiotic stress and environments.

|  | df | GY^†^ | AD | SD | ASI | EPP | HC | EA |
| --- | --- | --- | --- | --- | --- | --- | --- | --- |
|  |  | t ha^-1^ | days | days | days | no. | % | 1-5 |
| Low Nitrogen stress | | | | | | | | |
| Replication | 1 | 0.14 | 18.23*** | 104.73** | 40.52* | 0.02 | 0.10 | 0.01 |
| Genotype | 79 | 0.86*** | 7.89*** | 34.02*** | 25.93*** | 0.04*** | 0.43** | 0.04*** |
| F_1_ hybrids | 77 | 0.87*** | 9.70*** | 33.16*** | 26.61*** | 0.04*** | 0.43** | 0.04*** |
| GCA | 12 | 3.18*** | 32.79*** | 1.04 | 28.42** | 0.11*** | 1.04*** | 0.12*** |
| SCA | 65 | 0.44 | 5.44* | 0.31 | 2.11 | 0.03* | 0.32* | 0.03* |
| Error | 79 | 0.35 | 1.12 | 12.63 | 10.00 | 0.02 | 0.21 | 0.02 |
| %GCA SS |  | 57 | 53 | 38 | 71 | 42 | 45 | 38 |
| %SCA SS |  | 43 | 47 | 62 | 29 | 58 | 55 | 62 |
|  |  |  |  |  |  |  |  |  |
| Random abiotic stress | | | | | | | | |
| Replication | 1 | 13.98*** | 313.60*** | 532.90*** | 28.90*** | 0.04 | 11.28 | 9.75** |
| Genotype | 79 | 0.60 | 20.36** | 49.57*** | 14.27** | 0.06 | 266.36*** | 0.79 |
| F_1_ hybrids | 77 | 0.57 | 16.51* | 40.50** | 13.27*** | 0.05 | 269.63*** | 0.75 |
| GCA | 12 | 0.74 | 34.31*** | 102.44*** | 41.53*** | 0.09* | 586.70*** | 1.09 |
| SCA | 65 | 0.54 | 13.22 | 29.06* | 8.05 | 0.04 | 211.10* | 0.68 |
| Error | 79 | 0.49 | 10.12 | 20.86 | 7.91 | 0.06 | 126.36 | 0.68 |
| %GCA SS |  | 20 | 32 | 39 | 49 | 29 | 34 | 23 |
| %SCA SS |  | 80 | 68 | 61 | 51 | 71 | 66 | 77 |

^†^AD, day to anthesis; ASI, anthesis-silking interval; EA, ear aspect; EPP, ears per plant; GY, grain yield; HC, husk cover; SD, days to silking.

* Significant at the P < 0.05 level of probability.

** Significant at the P < 0.01 level of probability.

*** Significant at the P < 0.001 level of probability.
